# Psychometric properties of the German version of the Self-Image Scale (SIS-D)

**DOI:** 10.1371/journal.pone.0230331

**Published:** 2020-03-16

**Authors:** Jan Brederecke, Jennifer L. Scott, Martina de Zwaan, Elmar Brähler, Frank Neuner, Michael Quinn, Tanja Zimmermann

**Affiliations:** 1 Department of Psychosomatic Medicine and Psychotherapy, Hannover Medical School, Hannover, Germany; 2 School of Psychology, University of Tasmania, Hobart, Australia; 3 Medical Center, University of Leipzig, Leipzig, Germany; 4 Department of Psychosomatic Medicine and Psychotherapy, University Medical Center Mainz, Mainz, Germany; 5 Department of Psychology, Clinical Psychology and Psychotherapy, Bielefeld University, Bielefeld, Germany; Medical University Innsbruck, AUSTRIA

## Abstract

**Background:**

The Self-Image Scale is a self-report measure originally developed for use in women with cancer. Two subscales assess appearance satisfaction (self-acceptance) and perceptions of partners’ acceptance of their appearance (partner-acceptance). This study aimed to increase the Self-Image Scale’s utility by 1) confirming the two-factor structure of the German version of the Self-Image Scale, 2) testing measurement invariance across sex and age groups and validity, and 3) gathering general population normative data.

**Methods:**

Confirmatory factor analysis methods were used to examine the proposed two-factor model in a random sample of adults from the general German population (*N* = 1367). Measurement invariance, scale reliability, and validity were assessed.

**Results:**

The original factor structure and measurement invariance across sexes and age groups were supported. Women showed significantly lower self-acceptance than men. Adolescent and young adult women showed higher self-acceptance than senior women. For both sexes, partner-acceptance lowered across successive age cohorts. Internal consistencies were good.

**Conclusions:**

Results support the use of the German version of the Self-Image Scale in research and clinical practice. Research directions include validation in further diseases, collecting normative data across countries, and dyadic research, particularly exploring partner-acceptance across the life span.

## Introduction

A considerable body of psycho-oncology research indicates that the diagnosis and treatment of cancer can lead to significant body image and sexual concerns in both women and men [1–3]. Diminished body image and sexual problems can, in turn, have negative consequences on different facets of mental health like anxiety and depression [4, 5] and sense of self or wholeness [6]. Additionally, a reduced body image has been shown to negatively impact interpersonal relationships, work and social functioning and quality of life as a whole [7–9].

However, the extent of body image impairment and sexuality issues in cancer patients, relative to healthy people, is still not fully understood. This is due, in part, to a relatively small number of case-control studies that compared cancer patients or survivors with healthy control groups. For example, Lehmann, Hagedoorn, and Tuinman’s [3] systematic review found only 25 studies that compared cancer survivors and healthy control groups across different facets of body image. Furthermore, there was no clear evidence that body image in cancer survivors is worse than in the general population; almost half the studies found no difference between healthy and cancer populations, and three studies found a more positive body image in cancer survivors [3]. However, heterogeneity in the measures employed and aspects of body image assessed, reliance on experimenter derived measures in some cases, and methodological issues across most studies contributed to equivocal findings.

Another impediment to discerning the impact of cancer on body image and sexuality is that most measures used in psycho-oncology research were developed for use with a specific cancer population (predominately female breast cancer patients), precluding comparisons to other cancer types or to normative populations in the general community [10].

Further, none of the measures commonly used in the oncology literature assesses relational aspects of body image in the context of intimate relationships. Many cancer treatments can negatively impact factors important for romantic relationships, such as feelings of sexual attractiveness and sexual communication and functioning [11]. In women with cancer, perceptions of their partners' appraisals may be particularly important for adjustment as women’s perceptions of partners’ reactions to their post-surgery appearance predict women’s sexual, marital and emotional adjustment after breast cancer [12].

In the broader marital literature, dyadic experimental research has shown that body image also has a significant social dimension in that self-image is influenced by intimate relationships, especially for women [13]. In addition, women's perceptions of their partners’ satisfaction with their bodies can also be inaccurate and more critical or negative than partners’ actual appraisals [14]. Further, studies with both young and mid-life women in dating and marital relationships demonstrated that women who experience a larger discrepancy between their own perception of how they look, compared to their perception of their partner’s satisfaction with their body, are more likely to experience greater body dissatisfaction [15–17].

The growing area of research exploring body image in men with cancer suggests that men can also experience body image difficulties associated with diagnosis and treatment side effects, such as changes to sense of masculinity, sexual self-concept and functioning [2, 18–20]. Though the amount of studies on male body image in the broader body image literature is small relative to female body image research, there is evidence that men’s body image also has an intimate partner relational component [21]. For instance, one study, involving reports from both partners in a dyad, shows that there is a strong association between body image and relationship quality for both women and men [22]. Additionally, dyadic research suggests that psychological processes and perceived relationship quality in intimate relationships influence body image, dieting behavior [23] and sexual satisfaction in both men and women [22]. Unfortunately, research on body image in cancer patients and in community samples is hindered by the lack of measures that include assessment of perceived partner responses to appearance.

The Self-Image Scale [SIS; 13] was specifically designed to address this gap. It was initially developed for use with Australian women diagnosed with breast and gynecological cancer. Women with these diagnoses are at risk for developing body image disturbances as these types of cancers threaten parts of the female body perceived as an integral part of being a woman [24–27]. The SIS consists of two scales; the self-acceptance scale (6 items) which measures a woman’s satisfaction or acceptance of her body, and the partner-acceptance scale (5 items), which assesses women’s perceptions of her partners’ appraisals of her body. The consideration of the perceived partner-acceptance is unique to the SIS and allows for a more in-depth analysis of the women’s body image with regards to her intimate relationship. The first application of the German version of the SIS [SIS-D; 28] in women with breast cancer showed that individual and dyadic factors were differentially associated with self- and perceived partner-acceptance in women with breast cancer. In particular, it was shown that women’s age and depressive symptoms and men’s marital satisfaction were important predictors of self-acceptance. Whereas women’s age, female relationship satisfaction and perspective on common dyadic coping predicted women’s perceptions of their partner’s acceptance of their appearance. The results provided support for the hypothesis that individual and dyadic factors impact differently upon women’s body image after breast cancer. Moreover, the consideration of different aspects of body image–self-acceptance and partner-acceptance–is important.

However, body image research in oncology is insular to the extent that it is largely reliant on measures that are disease and sex-specific and developed and evaluated with older people, as cancer incidence increases with age [29]. They additionally lack normative data from the general population that can aid the interpretation of the findings by being able to describe differences to the general population. The clinical and research utility of the SIS-D would be enhanced by research aimed at overcoming these limitations.

### Study objectives

The current study thus aims to 1) confirm the two-factor structure of the German version of the SIS (SIS-D), 2) test measurement invariance across sex and age groups and, 3) gather general population normative data. Additionally, internal consistency and validity of the SIS-D in relation to Body Mass Index (BMI), depressive symptoms, and anxiety will be considered.

Regarding the normative data, it was expected that women would show lower scores on the self-acceptance scale than men, consistent with meta-analytic research findings that women overall have lower body image satisfaction than men [30, 31].

As for age effects on body image, no differences between younger and older women were expected, as the available literature indicates that women’s satisfaction with their bodies does not significantly alter with age [32]. The literature on the development of body image across the life-span in men is scarce and therefore, age effects on self-acceptance in men were investigated in an exploratory way. Further, there was no hypothesis regarding age or sex differences in perceived partner-acceptance due to a lack of literature in this area.

In order to assess validity, correlations of body image (SIS-D) with BMI, depressive symptoms, and anxiety were considered. Former studies have shown a negative correlation between body image facets, like body satisfaction, and BMI [33]. The same pattern was observed for correlations of body image and depressive symptoms in various settings [34–36]. Anxiety was also found to correlate negatively with body image facets such as body satisfaction [37]. Thus, it was hypothesized that self-acceptance would be negatively associated with BMI, and measures of depression and anxiety symptoms. No predictions were made in terms of perceived partner-acceptance and associations with BMI, depression or anxiety due to the paucity of research on intimate partner-related body image.

## Material and methods

### Data sampling

Data in this cross-sectional study were obtained from a representative sample of the German population regarding age, sex, and educational level. The survey was carried out by professional interviewers from a demographic consultation company (USUMA, Berlin, Germany). The participants had to be 15 years of age or older and speak German fluently. In order to assess the SIS partner-acceptance scale, participants in the present study needed to be in a relationship as well. Participants were chosen randomly with the help of a three-stage sampling procedure. In the first step, a selection of 258 regional sampling areas was randomly chosen; in the second step, a random procedure to select households of the respective areas was implemented; the final step was a pre-specified, standardized and random selection of one member of the selected household that fulfilled the inclusion criteria. All subjects were visited at home by a study assistant, if not at home, a maximum of three attempts was made to contact the selected person. Face-to-face interviews with the selected individuals were then conducted by the interviewers. Participants were informed in written form about the study and gave written informed consent. For underage participants, verbal parent or guardian consent was obtained by the interviewers and only then participation was possible. The next step was the assessment of sociodemographic data through the interviewers. Participants then completed a battery of self-report questionnaires. The study as well as the verbal consent procedure for the minor participant’s parents or guardians was approved by the Ethical Committee of Leipzig University, Germany (Az 452-15-21122015).

### Participants

Of the 1431 participants who took part in the survey, 95.5% (*N* = 1367) completed the SIS-D without missing values and were thus included in further analyses. 95.6% of the participants held German citizenship and the average age was 49.05 years (*SD* = 15.39, range = 16–89). The sample consisted of 713 women (52.2%) and 654 men (47.8%). Women were on average 47.25 years old (*SD* = 14.8, range = 16–89) while men were 51.01 years (*SD* = 15.78, range = 16–88) old on average. Men were significantly older than women in this sample, *t*(1365) = 4.53, *p* < .001. To test for measurement invariance across age, participants were assigned to three age groups: adolescents and young adults (AYA; 15 to 39 years old, *n* = 432); adults (40 to 67 years old, *n* = 768) and seniors (above the age of 67, *n* = 167). Age group thresholds were chosen according to the Adolescent and Young Adult Oncology Progress Review Group’s definition of AYA [38] and the usual German retirement age of 67 to ensure reasonable group sizes for the analyses and to enable back references to the field of psycho-oncology.

Age groups differed significantly in their levels of education with higher educational levels in the younger groups, χ^2^ (6, *N* = 1367) = 92.96, *p* < .001 (see Table 1). Regarding the distribution of the sexes, women were a small majority in the AYA and adult groups while men were the majority in the retired group, χ^2^ (2, *N* = 1367) = 20.16, *p* < .001 (see Table 1).

**Table 1:** Distribution of educational status and sex by age group.

		AYA	Adults	Seniors	Total
		(*n* = 432)	(*n* = 768)	(*n* = 167)	(*N* = 1367)
Education (n, %)					
	< = 9 years of school	68 (15.7%)	258 (33.6%)	89 (53.3%)	415 (30.4%)
	10 years of school	221 (51.2%)	328 (42.7%)	43 (25.7%)	592 (43.3%)
	>10 years of school	116 (26.9%)	156 (20.3%)	30 (18.0%)	302 (22.1%)
	other	27 (6.3%)	26 (3.4%)	5 (3.0%)	58 (4.2%)
Sex (n, %)					
	Male	187 (43.3%)	361 (47%)	106 (63.5%)	654 (47.8%)
	Female	245 (56.7%)	407 (53%)	61 (36.5%)	713 (52.2%)

Sums of more than 100% in a category result due to rounding to one decimal place; AYA = 15–39 years; Adults = 40–67 years; Seniors = >67 years.

### Measures

#### The German version of the Self-Image Scale (SIS-D)

The SIS-D is the German version of the SIS. The original translation of the SIS into German was done by Zimmermann, Scott, and Heinrichs [28]. The researchers translated the English version to German before it was translated back by a native English speaker. The resulting version of the SIS was then used to study individual and dyadic variables relevant for female cancer patients’ body image in a sample of German breast cancer patients and their spouses. For the current study, all femininity specific items were presented in a masculinity specific version as well (e.g., “I think my partner sees me as a woman/man”) in order to include men. The SIS consists of eleven items that are assigned to two scales: self-acceptance (six items) that assesses sense of femininity/masculinity as well as acceptance of appearance and self (e.g., “I like the way I look undressed”; “I feel good about myself”) and partner-acceptance (five items) that assesses participants’ perceptions of their partner’s responses to, and acceptance of their appearance (e.g., “I think my partner finds me sexy”; “I think my partner sees me as feminine/masculine”). The SIS asks for this evaluation regarding the past week. Items are rated on a five-point Likert-scale ranging from 1 (*strongly disagree*) to 5 (*strongly agree*). Two items are negatively formulated and thus reverse coded afterward. The self-acceptance scale score ranges from 6 to 30 while the partner-acceptance scale score ranges from 5 to 25. Higher scores suggest greater acceptance. Previous research suggests good psychometric qualities with internal consistencies [Cronbach’s α; 39] of .83-.92 for self-acceptance and .82-.91 for partner-acceptance [13] and α = .92 for the total scale in the German translation [28]. The SIS can be used to discriminate between healthy women and women newly diagnosed with breast cancer [13].

#### The German version of the patient health questionnaire 4 (PHQ-4)

Symptoms of anxiety and depression were assessed using the German version of the PHQ-4 [40]. The PHQ-4 consists of a 2-item depression scale [PHQ-2; 41] and a 2-item anxiety scale [GAD-2; 42]. The PHQ-2 is a short version of the PHQ-9 [43] a well-established screener for symptoms of depression. The GAD-2 is a short version of the GAD-7 [44] an equally well-established screening instrument for symptoms of anxiety. Items of the PHQ-4 correspond to symptoms of either major depressive disorder or generalized anxiety disorder in the Diagnostic and Statistical Manual of Mental Disorders diagnostic criterion A [DSM-IV-TR; 45]. They are rated on a four-point Likert-scale ranging from 0 (*not at all*) to 3 (*nearly every day*). PHQ-4 total subscale scores range from 0 to 6, and a cutoff score of 3 or higher indicates positive depressive or anxiety disorder screening [46]. The PHQ-4 showed good internal consistency in the original study [α = .85; 40] as well as in the present study (α = .86).

#### Body mass index

Weight and height were self-reported; the BMI ([(weight in kg) / (height in m)^2^]) was calculated.

### Statistical analysis

All statistical analyses were performed in *R* version 3.4.4 [47]. The R packages used for all confirmatory factor analysis (CFA) related calculations are *lavaan* [48] and *semTools* [49]. All tests were based on a significance level of .05 (if not stated otherwise).

#### Factor structure

Confirmatory factor analyses were conducted to test the proposed two-factor model of the SIS. Parameters of the CFA models were estimated with a weighted least squares estimator that uses diagonally weighted least squares as well as mean and variance adjusted test statistics as the SIS is measured on a categorical scale [WLSMV; 50]. Model fit was assessed using the following fit indices: χ^2^ test statistic for absolute fit, comparative fit index [CFI; 51] and Tucker-Lewis Index [TLI; 52] for fit relative to a null model, Root Mean Square Error of Approximation and 90% Confidence Interval [RMSEA; 53] as cited in Hu and Bentler [54] and Standardized Root Mean Square Residual [SRMR; 55] as cited in [54] for overall fit. The χ^2^ test statistic is sensitive to large sample sizes and thus tends to reject models in large samples [56]. According to Hu and Bentler [54], good model fit can generally be assumed when CFI and/or TLI is higher than 0.95 (>0.90 is acceptable), SRMR is smaller than 0.08 and RMSEA is smaller than 0.06 (<0.09 is acceptable).

#### Measurement invariance

To determine whether SIS-subscales could be meaningfully compared across sex and age-groups, measurement invariance was tested using multi-group confirmatory factor analyses. The analyses were performed using WLSMV method of estimation. Nested and increasingly invariant models of the SIS subscales were compared according to recommendations of Gregorich [57]. Configural, metric, and scalar invariance, were examined. The procedure will be described only briefly, for further information on measurement invariance testing see e.g., Vandenberg and Lance [58], Cheung [59] or Sass, Schmitt, and Marsh [60]. The configural invariance model is used as a baseline model for all successive models and assumes the same pattern of fixed and free loadings across groups. If configural invariance is not supported, the constructs differ substantially across the groups, and the more restricted models can neither be supported [58]. The metric invariance model assumes that the groups respond to the items in the same way. This is necessary in order to enable a meaningful comparison of scores across groups. Metric invariance is tested by fixing factor loadings to equality across groups and then comparing the fit of the metric invariance model to the fit of the configural invariance model. If the model fit worsens significantly, metric invariance is not supported. Models were compared using the method of Satorra [61] for scaled χ^2^ difference testing. In this case, only the resulting *p*-values are interpretable, so neither the difference in χ^2^ (Δχ^2^) nor in degrees of freedom is interpretable. Additionally, the difference in CFI (ΔCFI) was considered. According to Cheung and Rensvold [62], a ΔCFI smaller than -.01 indicates that two compared models can still be treated as invariant, even if the χ^2^ difference test is significant. Scalar invariance is a necessary prerequisite to compare means in latent constructs across groups meaningfully. When scalar invariance is established, differences in the means of the observable items can be attributed to differences in the means of the latent variable. It is tested by setting equal thresholds for the groups and then testing the model fit against the model fit of the metric invariance model. If the model fit worsens significantly, scalar invariance is not supported.

#### Group differences

Group differences were calculated using t-tests, ANOVA and Tukey’s honest significance test [Tukey HSD; 63] as a post-hoc test.

#### Validity and internal consistency

Additionally, Pearson correlations of the SIS subscales with the PHQ-4 depression and anxiety subscales as well as with the BMI were calculated in order to examine validity. Scale scores were treated as continuous variables according to recommendations of Harpe [64]. The internal consistency of the subscales was assessed by calculating the Cronbach’s α coefficient [39] for both subscales of the SIS-D.

## Results

### Factor structure

**Fig 1:**
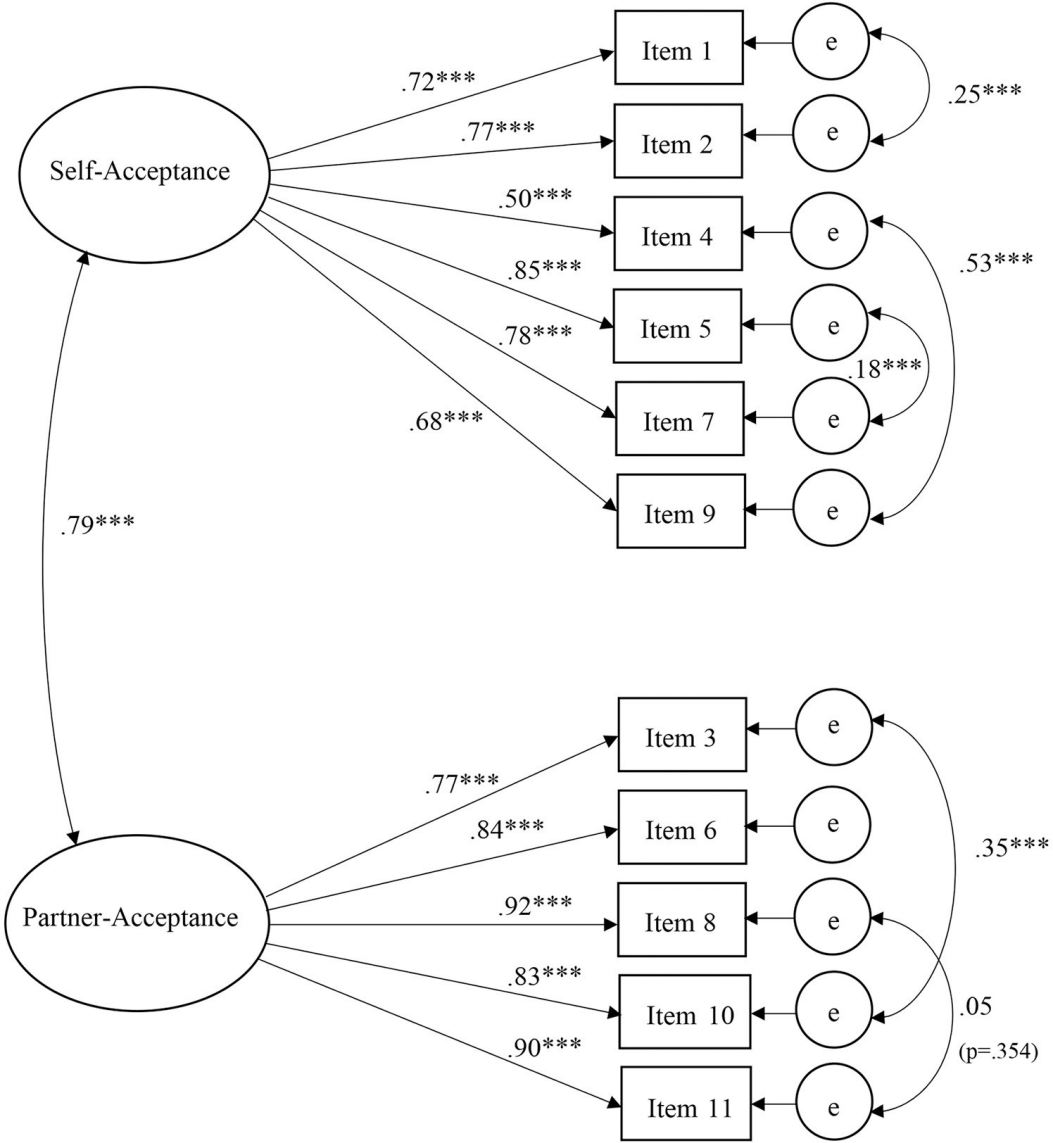
Standardized factor loadings, factor correlations and error covariances of the SIS-D. ***p < .001.

It was tested if the data fitted the originally proposed two-factor structure of the English version as developed by Scott et al. [13]. As shown in Table 2, this model did not fit the data well across all of the employed indices (e.g., RMSEA = .15). In order to improve model fit, modification indices were consulted. With a modification index of 348.51 (standardized expected parameter change = .35), including the error covariances of the two reverse-worded items 4 and 9 (θ_4,9_) showed the highest potential for model improvement. Combined with the very similar content of the items (Item 4: “I avoid looking at myself”; Item 9: “I dislike my appearance”), it was likely that the covariances of these items were not adequately taken care of by the latent variable. Furthermore, the possible negative effects of reverse-worded items on model fit and scale validity have been shown repeatedly in the past [65, 66]. Thus, the model was refit including the free error covariances of items 4 and 9. This resulted in a significant improvement in model fit (*p* < .001). Nevertheless, the RMSEA for the model stayed above the threshold for a good fit (.11). Another significant covariance (modification index = 56.80, standardized expected parameter change = .13) between items 3 and 10 was detected. The two items overlapped significantly regarding content as well (Item 3:” I think my partner sees me as a woman/man”; Item 10:” I think my partner sees me as feminine/masculine”). Including this covariance in the model led to another significant improvement in model fit (*p* < .001). Nonetheless, the model kept a high RMSEA (.11) while the other indices consistently showed a good fit for this solution. The wording of the remaining items revealed additional pairs of mutually overlapping items regarding their contents (Items 1&2, 5&7 and 8&11; see Table 3). Their freed covariances were also included in the fourth and final model in order to coherently represent this structure on model level. The final model improved once more in model fit (*p* < .001) without changing RMSEA significantly. A high correlation between the latent variables (*r* = .79) suggested the constructs self-acceptance and partner-acceptance are considerably overlapping but still beneath the threshold (*r* = .85) proposed by Cohen et al. [67]. The total sample CFA factor loadings of the final model are displayed in Table 3 (for more details see Fig 1). Standardized factor loadings ranged from .50 to .92 and were all statistically significant (*p* < .001). In sum, the results suggest the final two-factor model of the SIS-D is sound and representing the data well.

**Table 2:** Confirmatory factor analyses of the SIS-D: Overall model fit.

Model	*χ*^2^	df	Δχ^2^^a^	RMSEA	90% Confidence Interval	CFI	SRMR	TLI
	1349.18***	43	-	.15	.14-.16	.95	.06	.94
with θ_4,9_ free	764.27***	42	286.96***	.11	.11-.12	.97	.05	.97
with θ_4,9_, θ_3,10_ free	668.35***	41	79.17***	.11	.10-.11	.98	.05	.97
with θ_4,9_, θ_3,10_, θ_1,2_, θ_5,7_, θ_8,11_ free	610.47***	38	74.42***	.11	.10-.11	.98	.04	.97

^a^ As stated before, the Satorra [61] corrected Δχ^2^ is not interpretable, only the *p*-value is interpretable.

***p < .001; RMSEA = Root Mean Square Error of Approximation, cutoff = .06; CFI = Comparative Fit Index, cutoff = .95; SRMR = Standardized Root Mean Square Residual, cutoff = .08, TLI = Tucker Lewis Index, cutoff = .95.

**Table 3:** Items and factor loadings of the SIS-D two-factor model.

No.	Original item (German version)	Loading (standardized)^a^
Self-acceptance		
1	I like the way I look undressed. (Ich mag mein Aussehen, wenn ich unbekleidet/nackt bin.)	**1**^b^ (.72)
2	I like the way I look in my clothes. (Ich mag es, wie ich in meiner Kleidung aussehe.)	**1.08** (.77)
4	I avoid looking at myself. (Ich vermeide es, mich selbst anzusehen.)	**.70** (.50)
5	I feel attractive. (Ich finde mich attraktiv.)	**1.19** (.85)
7	I feel good about myself. (Ich finde mich gut.)	**1.08** (.78)
9	I dislike my appearance. (Ich mag meine Erscheinung nicht.)	**.95** (.68)
Partner-acceptance		
3	I think my partner sees me as a woman/man. (Ich denke, dass mein Partner/meine Partnerin mich als Frau/Mann sieht.)	**1**^b^ (.77)
6	I think my partner enjoys sexual intimacy with me. (Ich denke, dass mein Partner/meine Partnerin sexuelle Intimität mit mir genießt.)	**1.08** (.84)
8	I think my partner finds me attractive. (Ich denke, dass mein Partner/meine Partnerin mich attraktiv findet.)	**1.19** (.92)
10	I think my partner sees me as feminine/masculine. (Ich denke, dass mein Partner/meine Partnerin mich weiblich/männlich findet.)	**1.07** (.83)
11	I think my partner finds me sexy. (Ich denke, dass mein Partner/meine Partnerin mich sexy findet.)	**1.16** (.90)

^a^ All factor loadings were statistically significant (*p* < .001).

^b^ One factor loading per factor was set to 1 to scale the factor [48].

### Measurement invariance

After demonstrating an acceptable fit for the two-factor model in the total sample, measurement invariance analyses investigating similarity across sex and age groups were conducted. Single-factor models for the self-acceptance (Table 4) and the partner-acceptance (Table 5) scales were fitted, in order to inspect both scales separately.

**Table 4:** Self-acceptance scale (model fit summary).

Model/ Groups	Overall fit indices				Comparative fit indices			
	*χ*^2^	df	RMSEA	CFI	Model comparison	Δχ^2^	ΔCFI	*p*
*Sex*								
Configural	22.71	12	.09	.99	-	-	-	-
Metric	30.34	17	.06	1	2 vs 1	7.27	.00	.201
Scalar	76.21	34	.06	.99	3 vs 2	55.56	.01	***
*Age groups*								
Configural	22.52	18	.08	.99	-	-	-	-
Metric	30.64	28	.04	1	2 vs 1	9.57	.00	.479
Scalar	99.43	62	.05	.99	3 vs 2	59.46	.01	.004

***p < .001; RMSEA = Root Mean Square Error of Approximation, cutoff = .06; CFI = Comparative Fit Index, cutoff = .95; SRMR = Standardized Root Mean Square Residual, cutoff = .08, TLI = Tucker Lewis Index, cutoff = .95.

**Table 5:** Partner-acceptance scale (model fit summary).

Model / Groups	Overall fit indices				Comparative fit indices			
	*χ*^2^	df	RMSEA	CFI	Model comparison	Δχ^2^	ΔCFI	*p*
*Sex*								
Configural	28.18	6	.14	1	-	-	-	-
Metric	30.14	10	.09	1	2 vs 1	2.80	.00	.593
Scalar	39.17	24	.06	1	3 vs 2	14.34	.00	.424
*Age groups*								
Configural	31.05	9	.14	1	-	-	-	-
Metric	34.51	17	.07	1	2 vs 1	5.92	.00	.656
Scalar	111.51	45	.08	.99	3 vs 2	64.47	.00	***

***p < .001; RMSEA = Root Mean Square Error of Approximation, cutoff = .06; CFI = Comparative Fit Index, cutoff = .95; SRMR = Standardized Root Mean Square Residual, cutoff = .08, TLI = Tucker Lewis Index, cutoff = .95.

#### Self-acceptance

First, the configural model, which does not introduce equality constraints on parameter estimates across groups, was examined. The configural model for the sexes showed a mixed model fit with good CFI while the RMSEA was above the threshold of .06. The same applies to the configural model for age groups. Then the configural models were compared to the metric models that use equal loadings across the groups. No significant changes in *χ*^2^ were observed, neither for cross-sex comparison nor for age group comparison. Second, the scalar models that use equal loadings and equal thresholds were tested against the metric models. This comparison showed a significant difference in *χ*^2^ across sex (*p* < .001) as well as across age groups (*p* < .01). Nevertheless, the ΔCFI for both group comparisons was smaller than -.01 so that invariance could still be assumed according to Cheung and Rensvold [62]. Thus, measurement invariance across sex and age groups can be assumed for the self-acceptance scale.

#### Partner-acceptance

Again, the comparison of the configural and the metric models showed no significant difference in χ^2^ for cross-sex and cross-age group comparison. The same holds for the scalar model compared to the metric model across the sexes. Scalar and metric models across age groups did differ significantly in χ^2^, but ΔCFI was smaller than -.01. Hence, measurement invariance across sex and age groups can be assumed for the partner-acceptance scale.

In sum, measurement invariance across the sexes and age groups was demonstrated, suggesting the SIS performed similarly for all tested groups.

### Normative data for the SIS-D

SIS-D normative subscale scores by age group and sex are presented in Table 6.

**Table 6:** Normative subscale scores for the SIS-D by sex and age group.

	AYA	Adults	Seniors	Across all Ages
	(*n* = 432)	(*n* = 768)	(*n* = 167)	(*N* = 1367)
Self-acceptance (*M*, *SD*)				
Men	23.76 (3.53)	22.77 (3.53)	21.46 (3.43)	22.84 (3.59)
Women	22.68 (4.42)	22.10 (4.03)	20.90 (3.75)	22.20 (4.17)
Both Sexes Combined	23.15 (4.09)	22.42 (3.81)	21.26 (3.55)	22.50 (3.91)
Partner-acceptance (*M*, *SD*)				
Men	20.94 (2.64)	19.65 (3.23)	18.39 (3.26)	19.82 (3.19)
Women	21.27 (3.15)	19.80 (3.32)	17.15 (4.01)	20.07 (3.51)
Both Sexes Combined	21.12 (2.94)	19.73 (3.28)	17.93 (3.59)	19.95 (3.36)

*M* = mean; *SD* = standard deviation; AYA = 15–39 years; Adults = 40–67 years; Seniors = >67 years.

#### Sex differences in self-acceptance (overall and within age groups)

Overall, male participants showed only slightly higher values on the self-acceptance scale than female respondents resulting in a mean difference (Δ*M*) of .64, *t*(1359.6) = 3.06, *p* = .002. In the AYA and adult age cohorts, significant sex differences in self-acceptance were observed with AYA (Δ*M* = 1.08, *t*(429.09) = 2.83, *p* = .005) and adult men groups (Δ*M* = .66, *t*(765.9) = 2.44, *p* = .015) showing significantly higher self-acceptance scores than AYA and adult women. In contrast, in the senior age group, a different pattern was observed; men and women did not differ statistically significantly in their self-acceptance (Δ*M* = .56, *t*(116.15) = .96, *p* = .340).

#### Sex differences in partner-acceptance (overall and within age groups)

Across age groups, sex differences on the partner-acceptance scale were not significant, *t*(1364.9) = -1.42, *p* = .155, with Δ*M* = .26. No significant sex differences were observed for AYA (Δ*M* = .32, *t*(426.04) = -1.16, *p* = .246) and adult (Δ*M* = .14 , *t*(759.36) = -.60, *p* = .548) groups regarding partner-acceptance. Only senior men differed from senior women in that they had significantly higher partner-acceptance scores, *t*(105.45) = 2.05, *p* = .042, with Δ*M* = 1.24.

#### Female self- and partner-acceptance across age

Regarding age group, an ANOVA for the age groups in women (*F*(2, 710) = 4.73, *p* = .009) and additional Tukey HSD post-hoc tests revealed that the AYA women reported significantly higher levels of self-acceptance than the retired women (Δ*M* = 1.78, *p* = .008). In women’s partner-acceptance scores, all age groups differed significantly from each other, *F*(2, 710) = 40.75, *p* < .001 (AYA vs. Adults, Δ*M* = 1.47 *p* < .001; Adult vs. Retired, Δ*M* = 2.65, *p* < .001; AYA vs. Retired, Δ*M* = 4.12, *p* < .001).

#### Male self- and partner-acceptance across age

In men, all age groups differed significantly from each other in self-acceptance scores, *F*(2, 651) = 14.62, *p* < .001. Self-acceptance decreased across successive age cohorts (AYA vs. Adults, Δ*M* = .99, *p* = .005; Adult vs. Retired, Δ*M* = 1.31, *p* = .002; AYA vs. Retired, Δ*M* = 2.3, *p* < .001). The same pattern was observed for men regarding the partner-acceptance, *F*(2, 651) = 24.41, *p* < .001 (AYA vs. Adults, Δ*M* = 1.29, *p* < .001; Adult vs. Retired, Δ*M* = 1.27, *p* < .001; AYA vs. Retired, Δ*M* = 2.55, *p* < .001).

### Validity

Correlations of SIS subscale scores with PHQ-4 subscale scores and BMI were computed as indicators of convergent validity. The self-acceptance scale was negatively correlated with the PHQ-2 depression scale (*r* = -.33, *p* < .001), the GAD-2 anxiety scale (*r* = -.29, *p* < .001) as well as with BMI (*r* = -.27, *p* < .001). The same pattern applies to the partner-acceptance scale with negative correlations of *r* = -.28, (*p* < .001) with the PHQ-2 depression scale, *r* = -.24, (*p* < .001) with the GAD-2 anxiety scale, and *r* = -.16, (*p* < .001) with BMI.

### Internal consistency of the SIS-D

Cronbach’s α for the self-acceptance and the partner-acceptance scales were .82 and .89, respectively, suggesting good internal validity of the subscales in the total sample.

## Discussion

It is well established that body image is a crucial aspect of quality of life that predicts psychological and sexual adjustment in cancer patients and survivors. However, the field of psycho-oncology could be advanced by measures that are valid, psychometrically sound, applicable to both sexes and for which there is normative data from the general population. Further, there is a need in psycho-oncology research, and the body image research field more generally, to explore body image in age groups beyond the predominate samples of young college-age people, and to assess partner-related aspects of body image, as interactions in romantic relationships can shape body image and self-schema [68–70]. The SIS is a unique measure that assesses both self and partner-related body image and the current study sought to increase the clinical and research utility of the German version of the SIS (SIS-D) by examining the two-factor structure, measurement invariance and validity of the SIS-D in the German general population as well as establishing normative data in both sexes and age groups across the life span.

### Factor structure, measurement invariance, internal consistency, and validity

CFA results show that the SIS-D adequately represents the two factors self-acceptance and partner-acceptance in all subgroups and has acceptable psychometrics though the test of the factor structure of the SIS-D turned out to be more challenging than expected. In order to arrive at a well-fitting CFA model, modification indices were consulted, and multiple correlations between reverse worded and similarly worded items were included. Ultimately, the identified model confirmed the two-factor structure proposed by Scott et al. [13], reflecting self-acceptance and partner-acceptance as two distinct constructs. The two factors are highly correlated but can still be treated as representing two distinct constructs [67]. This differentiation is essential for the SIS-D’s value as a screening tool especially in clinical practice, as the partner-acceptance scale’s results can be used as a low-threshold entry point into the exploration of topics like relationship quality and sexuality, vital but often difficult topics to broach in psycho-oncologic care [71].

The SIS-D showed good psychometric properties. Configural, metric and scalar invariance were tested according to Gregorich [57], and it appears that similar concepts of self-acceptance and partner-acceptance exist across the sexes and age groups when Cheung and Rensvold’s [62] approach for evaluation is applied. While the SIS-D was developed as a questionnaire for women with cancer, the results of this study suggest, that men and women of different age interpret the self- and partner-acceptance constructs quite similarly. The SIS-D seems thus fit for use in the general German population as well as in cancer patients.

The SIS-D subscales showed good internal consistency and the negative correlations with the BMI, depressive symptoms and anxiety were consistently observed as expected from the literature [72].

### Normative subscale scores

#### Self-acceptance

As hypothesized, women overall showed lower self-acceptance than men. The result is consistent with meta-analytic research findings that women generally report lower body satisfaction compared to men [30, 73]. However, the size of this difference in the overall sample, though statistically significant, was small and thus its clinical relevance is questionable. The smaller difference than one might anticipate from the literature could, in part, be due to the dimension of body image assessed by the SIS self-acceptance scale. Research that reports larger sex differences in body image has tended to focus on satisfaction with body parts, shape, weight, and thinness, perceptions of muscularity/femininity, and eating disorder symptoms [30, 73]. In contrast, the SIS self-acceptance scale assesses feelings about overall appearance, attractiveness, and self-esteem.

However, the examination of sex differences in the entire sample masks age-related sex-differences in self-acceptance. The largest sex difference was apparent in the younger age group; young men shower higher self-acceptance than young women. This is consistent with findings from meta-analytic research [73] reviewing 50 years of research that was mainly conducted with young people, indicating that males report higher self-ratings of their attractiveness and satisfaction with their bodies than females report [74].

In the current study, self-acceptance lowered in incrementally larger magnitudes across each successively older age group for both men and women. Thus, the size of the sex differences in self-acceptance diminished across successive age groups and differences were not statistically significant in the senior age group. The reduction in self-acceptance scores across age cohorts was largest for men. Interpreting these results is difficult as there is a paucity of body image research conducted with middle-aged and elderly women, or with males generally (beyond dimensions traditionally related to muscularity) or across the life span [75]. In the current study adolescent and young adult (AYA) women showed higher self-acceptance than senior women. We had predicted that there would be no differences between younger and older women as some research indicated that women’s satisfaction with their bodies remains stable as women age [32]. While there is some evidence that women become more accepting of changes in aspects of their physical appearance as they age [76], age-related changes can contribute to women’s sense that their appearance does not match societal ideals of the youthful, wrinkle-free and vital female form, with concomitant feelings of loss of attractiveness [77, 78]. Research with older women has tended to focus on body dissatisfaction, not general feelings of attractiveness and overall appearance satisfaction as measured by the SIS [79, 80].

To our knowledge, this is the first study to examine male body image that relates specifically to acceptance of overall appearance, attractiveness, and self-esteem. The limited body image research in men suggests that, whilst it is multifaceted, it can include appraisals of dimensions that are different to women (and that the SIS self-acceptance subscale does not capture), such as perceptions of muscularity, height, penis size and body hair distribution [75]. The current results suggest that appearance esteem, as assessed by the SIS-D, is lower in middle-aged and older men compared to younger men, and that sex differences in self-acceptance between middle-aged and elderly men and women are small to negligible.

#### Partner-acceptance

For both sexes, partner-acceptance was lower in each successive age cohort and was lowest in senior men and women. Men and women did not differ statistically in their partner-acceptance scores overall, only within the senior age cohort with women showing lower scores. The SIS partner-related subscale is comprised largely of items related to sexual attractiveness and enjoyment of sexual intimacy. Thus, while people might accept their own appearance, they may believe their partners do not find them sexually attractive, especially in the older age group. Changes to bodily functions, appearance, and health associated with aging processes can negatively impact of sexual functioning in both men and women [76], and can lead to loss of muscularity and feelings of sexual virility in men, or to feelings in women that they do not match the social value placed on attractiveness for women that includes the lithe, youthful and sexy femme fatale attributes portrayed in media [78]. Consequently, older men and women might come to believe that their partners find them less sexually attractive. There is some evidence from dyadic research using corroborating objective assessment methods that people in romantic relationships are fairly accurate in their judgments of some partner and relationship domains [81]. In the current research, whether such appraisals are accurate cannot be determined as partners’ reports were not obtained. Further, whether people regard their partners’ views of them as less sexy as being problematic was not assessed; there is evidence that intimacy in older couples is not heavily dependent upon sexual thoughts and sexual activity and instead associated with emotional intimacy [82].

### Limitations

There is a number of limitations to the study that need to be considered. First, the data is based entirely on self-report measures and the study design is cross-sectional; these methods can pose issues that are common in psychological research, namely response bias and common method variance [83].

Moreover, despite its size, the sample is not necessarily representative in terms of the distribution of nationalities. With a share of 95.6% of persons with German citizenship, persons with non-German citizenship are underrepresented in the present sample as their share should be around 13% [84].

Further, researchers were present when participants completed the questionnaires, and this may have increased socially desirable responding and thus the extent of self- and/or partner perceived acceptance may have been inflated. Reports were only obtained from one partner in the dyad precluding assessment of the accuracy of reported partner-related perceptions and the cross-sectional design means age and cohort effects are difficult to disentangle and interpret. Research rigor could be improved by dyadic longitudinal designs that explore self- and partner-acceptance across the life span and that collect data from both partners. A number of variables can influence the accuracy of judgments in romantic relationships such as depression and self-esteem and should serve as control variables.

In addition, body image, sexual satisfaction, closeness and intimacy, and accuracy in judgments of partner behaviors can be influenced by relationship factors such as relationship satisfaction, quality, and length [22, 85]. Responses to the partner-related subscale may be influenced by whether participants are sexually active/intimate with their partner as well. To aid interpretation of data these relational variables should be assessed in future research.

Participants’ physical condition and whether they had a history of, or current body image or eating disorders, were not assessed and reports from people with physical health problems/diseases, disability scars, body image or eating disorders may have influenced results and should be controlled in future studies. Given the high correlation between the subscales in the current sample, convergent and divergent validity needs to be explored to a greater extent in future research. The relationship between the SIS-D subscales and sexual self-schema, other measures of body image and discriminate validity between health and illness samples should be examined.

Additionally, age group thresholds were based upon classifications used in a psycho-oncology review of health outcomes in adolescents and young adults with cancer, commissioned by the American National Cancer Institute [38]. However, to facilitate age group comparisons across normative populations and body image research, future research should employ age cut-offs that either align with conventions in body image research [32, 86] or that are informed by developmental stages in psychology research such as those generally used by the WHO [87].

Finally, people above the age of 67 were underrepresented in this sample. The smaller number relative to the other age cohorts means results for the older group need to be interpreted with caution, although the current sample is moderately large compared to other body image studies involving older people [76].

### Strengths

The results contribute significantly to the field of psycho-oncology and body image research more broadly. First, to our knowledge, the SIS is the only body image measure for use in the general population in the context of intimate relationships that assesses perceived relational aspects of body image, particularly men's and women’s perceptions of their partners’ views about their appearance and sexual attractiveness. In the body image field, the *Tripartite model of body dissatisfaction* [88] has informed research that predominately focuses on the influence of peers, parents, and media. The role of romantic partners in shaping body image has been examined in the context of eating disorders but the assessment of the relational aspects of body image in non-clinical populations has received considerably less attention. Currently, there is one other published, validated body image measure that assesses partner-specific behavior; the modified Verbal Commentary on Physical Appearance Scale [VCOPAS; 89]. The modified VCOPAS was developed and validated with college-age women and assesses females’ recall of the frequency of romantic partners’ negative and positive comments about weight/shape. The modified VCOPAS does not assess important relational aspects of their partners’ responses, such as comments about femininity, sexual intimacy, and sexual attraction. Further, the modified VCOPAS does not assess the inferences women make about partner comments; perceptions of partner behavior can be stronger predictors of individual and relational outcomes than actual or recalled enacted behavior [90].

This is the first study to directly compare sex differences in partner-specific relational aspects of body image in the general population. The results suggest that men and women are similar in beliefs about partners’ views of aspects of their sexual selves.

Few studies have examined male body image and the current findings extend knowledge in the field. Middle-aged and older men were found to report lower satisfaction with their overall appearance and feeling of attractiveness than younger men; a pattern of results that is replicated in the women in the sample.

The acquired population data allows for the use of the SIS-D in the general population and makes it thus more broadly applicable.

### Clinical implications

The results of the current study suggest that the SIS-D is a useful tool for psycho-oncological practice. Its short length makes it a valuable screening tool that can easily be incorporated in routine assessments in the psycho-oncologic setting. Both subscales can be used to assess cancer patients in romantic relationships whilst using only the self-acceptance subscale could be appropriate for patients not in a relationship. Through comparison with normative data, clinicians can gauge the extent of the impact of cancer experience on body image disturbance to determine if intervention is warranted. Clinicians might also use patient’s SIS-D results as a starting point for the exploration of sensitive topics like partner-acceptance, intimate relationship quality or sexuality and to assess changes in self- and partner-acceptance over time in response to either medical treatment or psychological intervention. Moreover, the use of the SIS-D in persons with other medical conditions (e.g. transplantation, heart disease, diabetes, multiple sclerosis) might also be useful but further research in this field is necessary. As this study provides general population data, the use in the (German) general population and in clinical populations, such as eating disorders is also reasonable when body image, sexuality, and sequelae are the research focus.

## Conclusions

The psychometric properties of the SIS-D indicate that it is a reliable and measurement invariant instrument for assessing self-acceptance and partner-acceptance aspects of a person’s body image. The differentiation of self- and partner-acceptance, although statistically closely related, is practically important as clinicians can sometimes overlook the importance of patients’ perception of partners’ reactions to their body and sexual intimacy, especially in older couples [91, 92]. Future research directions include validation in male patient samples and in a range of female cancers (beyond breast and gynecological cancer tested in the SIS development) as well as other medical conditions and amassing normative data for a range of nations to enable cross-country comparisons.

## Supporting information

S1 Dataset This SPSS dataset was used for all calculations in the present study. (SAV)
